# Numerical Study of Contact Behavior and Temperature Characterization in Ultrasonic Welding of CF/PA66

**DOI:** 10.3390/polym14040683

**Published:** 2022-02-11

**Authors:** Yuanduo Yang, Zhiwei Liu, Yuefang Wang, Yang Li

**Affiliations:** 1 School of Materials Science and Engineering, Tianjin University, Tianjin 300354, China; yyd111865@tju.edu.cn; 2 Tianjin Key Laboratory of Advanced Joining Technology, Tianjin University, Tianjin 300354, China; 3 Department of Engineering Mechanics, Dalian University of Technology, Dalian 116024, China; liu199224@126.com (Z.L.); yfwang@dlut.edu.cn (Y.W.)

**Keywords:** ultrasonic welding, carbon fiber reinforced thermoplastic (CFRTP), harmonic balance method (HBM), heating generation, welding parameters

## Abstract

Ultrasonic plastic welding (UPW) is a promising method for joining carbon fiber reinforced thermoplastic (CFRTP). The interface temperature determines weld quality to a large extent. This paper numerically analyzes the contact behavior and temperature characterization during welding using harmonic balance method (HBM). The simulation and experimental results show that amplitude and welding time are important factors determining the interface temperature. Increasing amplitude and welding time can significantly increase the interface temperature. Plunging speed and trigger force have little effect on the interface temperature. For nonlinear contact and heat generation, the results show that there is a certain separation between workpieces and the heat source is mainly friction heat generation in the early stage of welding. With the progress of welding, there is no separation between the workpieces and viscoelastic heat generation begins to dominate.

## 1. Introduction

Lightweight materials can effectively reduce fuel consumption and exhaust emissions in the transportation domain, which is of great significance for environmental protection [1,2]. Carbon fiber reinforced thermoplastic (CFRTP) is increasingly favored by automobile and aerospace industries due to its superior mechanical properties (high specific modulus and specific strength), good weldability, short production cycle and potential recyclability [3,4,5]. As a promising joining method for CFRTP, ultrasonic plastic welding (UPW) has the advantages of high efficiency, energy saving, easy to operate and convenient for in-situ monitoring [6,7,8,9]. In recent years, the development of ultrasonic sequential welding and ultrasonic continuous welding has greatly promoted the application of ultrasonic welding technology in the assembly of large CFRTP parts [10,11,12].

The heat generation and temperature characterization in ultrasonic CFRTP welding have important impacts on the weld quality. Many researchers have carried out a lot of effort on it, which has revealed some basic rules in the process. However, due to the complexity of the process, there is still insufficient cognition in the heating mechanism and temperature evolution. Numerical simulation is a powerful tool to investigate the UPW process. Establishing an efficient and accurate numerical simulation method can help to deepen the understanding of this process.

As early as 1980s, Frankel and Wang [13] studied the energy transfer and joining mechanism of UPW and developed a simple model to predict the temperature rise of the weld interface below the glass transition temperature. Khmelev et al. [14] modeled UPW from the perspective of wave propagation and calculated the distribution of input energy in the weld area. Suresh et al. [15] modelled the UPW process based on ANSYS for various thermoplastic materials and obtained the corresponding temperature distribution. However, the change of storage modulus and loss modulus of materials with temperature were not considered in their research. Zhang et al. [16] analyzed the heat generation mechanism of UPW and found that the friction heat generation at the interface was the main heat source before viscoelastic heat generation began to dominate. Wang et al. [17] constructed a finite element model based on the viscoelastic dissipation theory to analyze the influence of the size and top angle of energy directors on the heat generation in UPW and found that the influence of the top angle of energy directors on the heat generation is much greater than that of their size. Levy et al. [18,19,20] conducted systematic numerical investigations on the UPW process and confirmed the friction heat generation at the interface is the main source of heat in the initial stage of welding and viscoelastic heat generation is dominant after the interface temperature reaches the glass transition temperature.

At present, there is still a lack of understanding on the contact behavior and temperature characterization during UPW. The authors have previously proposed a numerical methodology based on harmonic balance method (HBM) that can reveal the contact behavior and temperature characterization in ultrasonic metal welding (UMW) and ultrasonic metal/CFRTP welding [21,22]. In UPW, the vibration direction of the horn is perpendicular to the workpieces while it is parallel with the workpieces in UMW. The heating mechanism in UWM is mainly frictional heating while UPW consists of viscoelastic heating and frictional heating. Due to the differences between UPW and UMW, this paper adopts a similar framework to the one in Refs. [21,22] but modified program codes for the simulation of ultrasonic CFRTP welding. The findings offer insights into the contact behavior and temperature characterization in ultrasonic CFRTP welding.

## 2. Numerical Methodology

### 2.1. Physical System and the Numerical Analysis Procedure

**Figure 1:**
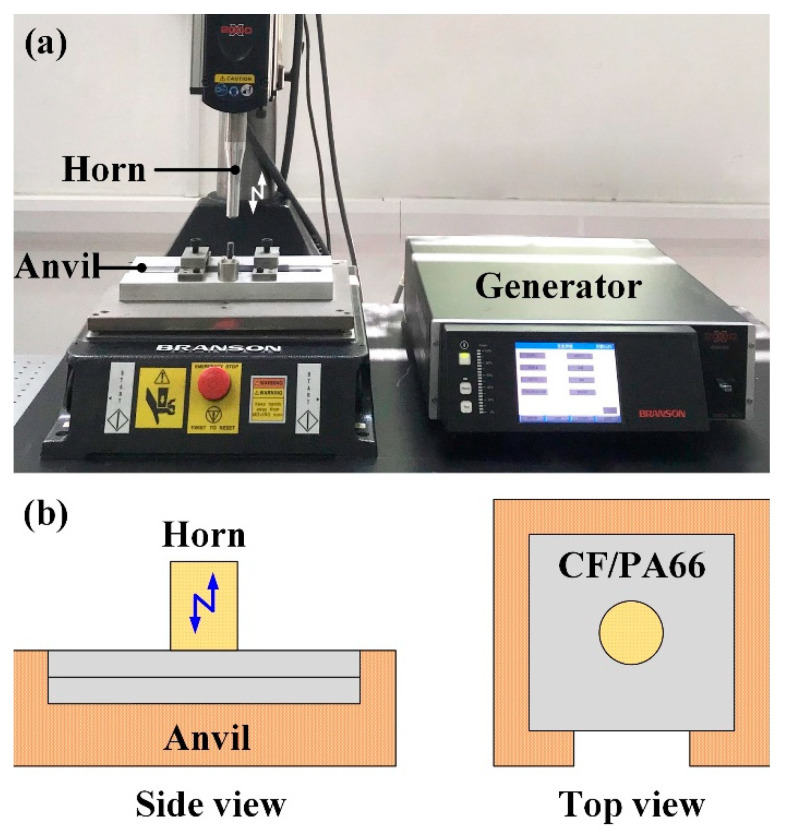
The (**a**) physical system; and (**b**) schematic diagram of UPW.

Figure 1 is the schematic diagram and physical system of UPW. The dimensions of workpieces were 40 mm × 40 mm × 3 mm.

**Figure 2:**
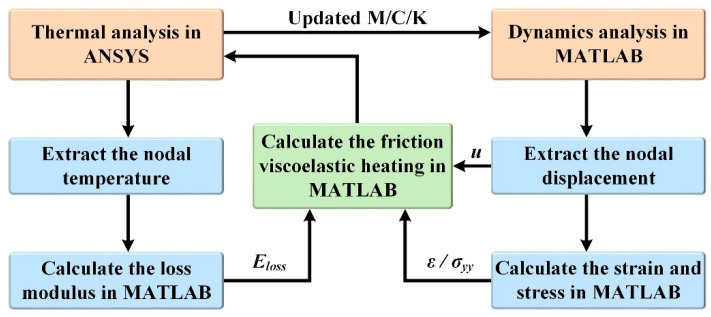
Procedure of the thermal-mechanical coupling computational method.

The authors have previously proposed a numerical method for the thermal-mechanical coupling analysis of nonlinear friction behavior and temperature characterization in ultrasonic welding of Al to Cu and Al to CFRTP [21,22]. A similar but modified computational approach is adopted in this paper to address the viscoelasticity of the CFRTP and the vertical ultrasonic vibration. The modified computational procedure is shown in Figure 2.

This method performs thermal analysis and mechanical analysis in ANSYS and MATLAB respectively. According to the calculated friction heat generation and viscoelastic heat generation, mass matrix (**M**), damping matrix (**C**) and stiffness matrix (**K**) are updated in ANSYS, and then MATLAB performs dynamic analysis according to updated **M**/**C**/**K**. The strain required for viscoelastic heat generation and the stress required for friction heat generation can be calculated according to the displacement obtained through dynamic analysis in MATLAB. The loss modulus required to calculate viscoelastic heat generation can be calculated according to the node temperature extracted by thermal analysis in ANSYS. The above process is carried out in cycles until the end of welding.

### 2.2. Structure Dynamics

The structural dynamic equations considering the nonlinear contact force of the workpieces can be expressed as [21,22]

(1) $$M\ddot{x}+C\dot{x}+Kx=f_{E}+f_{C}(x),$$

where **M**, **C** and **K** are the mass, damping and stiffness matrix, respectively; **x** denotes the displacement vector, **f*_E_*** represent the external force vector, and **f*_C_*** represent the nonlinear contact force vector. This equation is discrete and N dimensional. The governing equation of a single-degree-of-freedom linear system can be expressed as:

(2) $$m\ddot{x}+k_{t}x=f,$$

where *m* is the mass, *x* is the displacement, *k_t_* is the stiffness, and *f* is the periodic external force with a frequency *ω*. The complex stiffness is composed of the real stiffness provided by the storage modulus *k_storage_* and the imaginary stiffness provided by the loss modulus *k_loss_*, i.e.,

(3) $$k_{t}=k_{storage}+ik_{loss}.$$

Substituting Equation (3) into Equation (2) for a harmonic excitation and response, one obtains

(4) $$m\ddot{x}+k_{storage}x+ik_{loss}x=f\;\mathrm{or}\;m\ddot{x}+k_{storage}x+\frac{k_{loss}}{\omega }\dot{x}=f,$$

where *k_loss_*/*ω* represents the damping coefficient. Modeling energy losses as proportional damping, one can define an equivalent proportional damping factor *η* as

(5) $$\frac{k_{loss}}{\omega }=\eta k_{storage}\;\;\mathrm{or}\;\;\eta =\frac{k_{loss}}{\omega k_{storage}}=\frac{E_{loss}}{\omega E_{storage}}.$$

### 2.3. Nonlinear Contact Model

**Figure 3:**
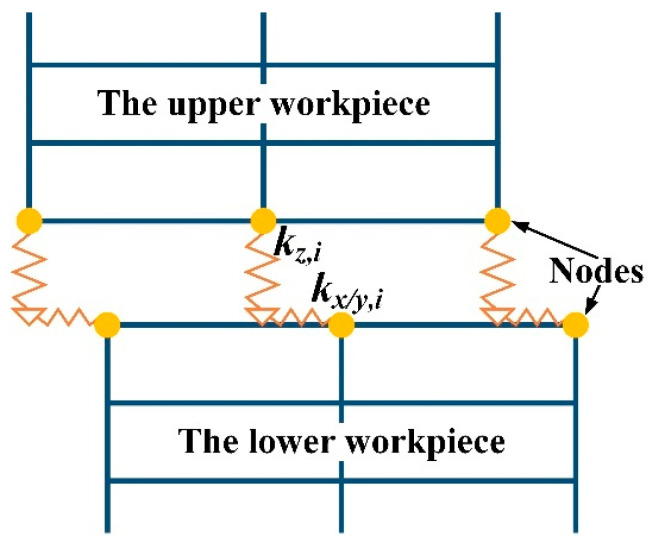
Schematic diagram of the nonlinear contact model [21,22].

Under the action of welding force, two workpieces are considered close contact. The contact is considered piecewise linear as it can only sustain compressive loads. When the horn is vibrating perpendicularly, the upper workpiece is vibrating together with the horn. Generation of friction heating occurs due to slight sliding between the workpieces in the early stage of welding. Figure 3 shows a schematic diagram of the nonlinear contact model [21,22], the parameters *k_x_*_,*i*_, *k_y_*_,*i*_ and *k_z_*_,*i*_ respectively represent the contact stiffnesses in the *x*, *y* directions parallel to the welding interface and *z*-direction perpendicular to the welding interface. The relative motions in the *x*, *y* and *z* directions are denoted by *u_x_*_,*i*_(*t*), *u_y_*_,*i*_(*t*), and *u_z_*_,*i*_(*t*), respectively. The normal force *N_i_*(*t*) is given by

(6) $$N_{i}\left(t\right)=\max\left(k_{z,i}u_{z,i}\left(t\right),0\right).$$

When the normal relative displacement (the difference between the node displacement of the upper workpiece interface and the lower workpiece interface) is positive, the two workpieces are in contact and the normal force is also positive. When separation occurs at the interface, the relative displacement is negative and the normal force is zero. The tangential friction forces in the *x* and *y* directions in various states of contact are given by

(7) $$T_{x/y,i}\left(t\right)=\left\{ \begin{array}{l} k_{x/y,i}\left(u_{x/y,i}\left(t\right)-w_{x/y,i}\left(t\right)\right)\;\mathrm{stick}\\ \mathrm{sign}\left({\dot{w}}_{x/y,i}\right)\mu N_{i}\left(t\right)\;\;\;\;\mathrm{slip}\\ 0\;\;\;\;\;\;\;\;\;\;\;\;\;\;\mathrm{separation} \end{array}\right..$$

### 2.4. Calculation of Heating Generation 

#### 2.4.1. Viscoelastic Heating

Tolunay et al. [23] deducted the viscoelastic heating in ultrasonic polymer welding:

(8) $$Q_{vis}=\frac{\omega \varepsilon^{2}E_{loss}}{2},$$

where *ω* is the vibration frequency, *ε* is the amplitude of dynamic strain. *E_loss_* is the loss modulus, which can be determined by the calculated nodal temperature and the temperature–loss modulus relationship obtained from a dynamic mechanical analysis (DMA) test. The sample dimensions for DMA tests were 37 mm × 10 mm × 1 mm. The frequency–temperature sweep method was used with the temperature range of 25~80 °C and the frequency range of 0.05~10 Hz. The displacement *u* at each node solved from Equation (1) is used to obtain the dynamic strain *ε* at each node by a standard process (in ANSYS) using the strain matrix **B** as *ε* = **B***u*. Thus, the amplitude of dynamic strain can be obtained by *ε* = *|ε|*.

#### 2.4.2. Friction Heating

The friction heat generation is another heat generation mechanism besides viscoelastic heat generation. Under the action of ultrasonic vibration, there is a phase difference between the upper and lower workpieces, which makes a small amount of sliding between workpieces. This relative sliding will cause friction heat generation. For UPW without an energy director, the friction heat generation can be described by the following formula [20,24]:

(9) $$Q_{fric}=\frac{\omega }{\pi }\mu |\sigma_{yy}(x)u(x)|,$$

where *μ* is the friction coefficient, *σ_yy_*(*x*) is the vertical stress on the interface, and *u*(*x*) is the horizontal displacement of the interface. 

### 2.5. Solution Using the Harmonic Balance Method (HBM)

The HBM is a commonly used method to determine periodic motions in nonlinear dynamics and the detailed algorithms and solution processes have been presented in literature [21,25] Therefore, only a brief description is given below.

To apply the HBM, the workpiece displacements, the external forces, and the contact forces between the workpieces are considered periodic in time and are expressed as a sum of harmonics [21,22,25]

(10) $$\begin{array}{l} x=ℜ\left\{\sum_{n=0}^{\infty }{\hat{x}}^{\left(n\right)}e^{in\omega t}\right\}\\ f_{E}=ℜ\{\sum_{n=0}^{\infty }{\hat{f}}_{E}{}^{\left(n\right)}e^{in\omega t}\}\;\;\;.\\ f_{C}=ℜ\left\{\sum_{n=0}^{\infty }{\hat{f}}_{C}{}^{\left(n\right)}\left(q\right)e^{in\omega t}\right\} \end{array}$$

Substituting Equation (10) into Equation (1), one obtains

(11) $$\sum_{n=0}^{\infty }\left\{\left[-{\left(n\omega \right)}^{2}M+in\omega C+K\right]{\hat{x}}^{\left(n\right)}-{\hat{f}}_{E}{}^{\left(n\right)}-{\hat{f}}_{C}{}^{\left(n\right)}\right\}e^{in\omega t}=0.$$

The solution process has been presented in Ref. [21], and is omitted here for the sake of brevity.

### 2.6. Heating Conduction

The governing equation for heat conduction can be expressed as:

(12) $$\rho c\frac{\partial T}{\partial t}=k\left(\frac{\partial^{2}T}{\partial x^{2}}+\frac{\partial^{2}T}{\partial y^{2}}+\frac{\partial^{2}T}{\partial z^{2}}\right)+S,$$

where *ρ* is the material density, *c* is the specific heat, *k* is the thermal conductivity, and *S* is the sum of frictional and viscoelastic heating. The temperature-dependent stiffness and damping matrices of the structure will be updated based on the calculated temperature.

Since the time scale (1 s) of heat conduction in UPW is significantly longer than the time scale (10^−4^ s) of the structural vibrations, the thermal field can be regarded as quasi-static compared with the vibrations, and the structure will reach a periodic quasi-steady-state vibration quickly at each temperature level. During each time period the structural vibrations are assumed to quickly reach a steady state, and hence the heat generated over time can be obtained from the HBM solution.

## 3. Experiments and Numerical Simulations

### 3.1. Experiments

**Figure 4:**
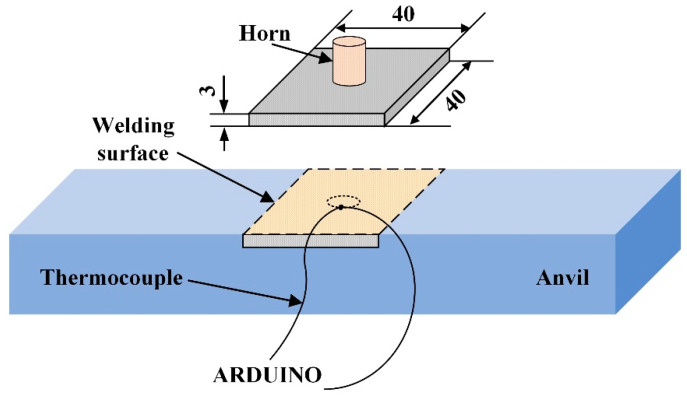
Schematic diagram of experimental setup and temperature measuring point (unit: mm).

The materials used in this research were 3-mm-thick carbon fiber reinforced PA6 (CF/PA6) composite (GCL-3H), provided by EMS-CHEMIE (Suzhou) Ltd. The mass fraction of carbon fiber was 30%. Figure 4 shows the schematic of the experimental setup. The temperature at the projection of the horn edge on the welding surface was measured using a K-type thermocouple with diameter of 0.125 mm. Arduino^®^ was used to record the temperature at a sampling frequency of 100 Hz.

A Branson 2000XD ultrasonic welder with a vibration frequency of 20 kHz was used to perform the welding. The welding parameters are as follows: amplitude 35 µm, trigger force 200 N, plunging speed 0.3 mm/s and welding time 0.5 s. Before welding, the CF/PA6 sheets were dried in an oven at a temperature of 70 °C for 24 h to remove moisture.

### 3.2. Finite Element Modeling

**Figure 5:**
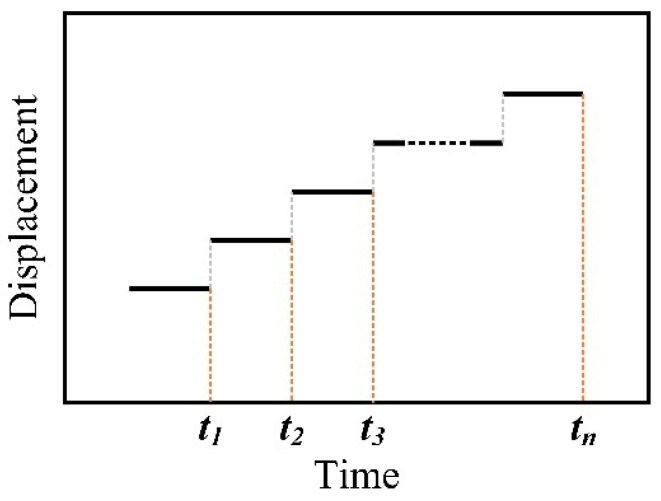
The horn displacement with time.

From the perspective of energy, the UPW system converts electrical energy into mechanical energy and then converts mechanical energy into thermal energy. For UPW, the horn moves downwards while it vibrates perpendicular to the workpiece interface. Therefore, it can be considered that the static displacement of the horn changes step by step in the calculation, that is, the static displacement of the horn remains unchanged in a short time and the vertical vibration takes this position as the equilibrium position, as shown in Figure 5. When the frequency domain method based on HBM is used to analyze the structural periodic response, only the first-order harmonic is selected as the harmonic term, because the proportion of subharmonic term and super harmonic term in the response can be ignored.

The welding parameters mentioned in this paper mainly include the welding amplitude, trigger force, welding speed and welding time. The specific numerical simulation scheme is shown in Table 1. The first of these gives the optimal parameters from the previous experiments, so this one is used as the base to study the effect of welding parameters on heat generation by changing each parameter separately.

**Figure 6:**
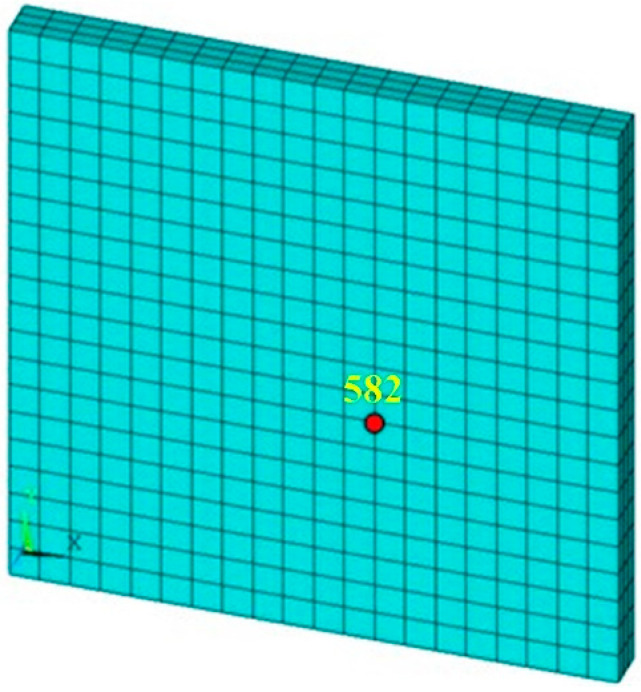
Finite element model of the CF/PA6 workpiece.

In this paper, ANSYS is used to construct the three-dimensional finite element model. Solid185 (structural field) and solid70 (temperature field) elements are used to mesh. When meshing, three elements are divided in the thickness direction to ensure accuracy and the total degree of freedom of two workpieces is 9261. The model of a single CF/PA6 composite workpiece is shown in Figure 6. In terms of boundary conditions, it is assumed that all contact points are consistent with the movement of the horn. Since the lower workpiece is supported by the anvil, it is assumed that all nodes on the lower surface of the lower workpiece are fixed. The whole welding process is divided into 10 steps according to length of time. During numerical simulation, it is assumed that the horn only vibrates in each small time period, that is, the horn shows the characteristics of step falling and vibration to be close to the reality.

Table 2 shows the materials properties that used in the simulation.

## 4. Results and Discussion

### 4.1. Contact Behavior at the Welding Interface

**Figure 7:**
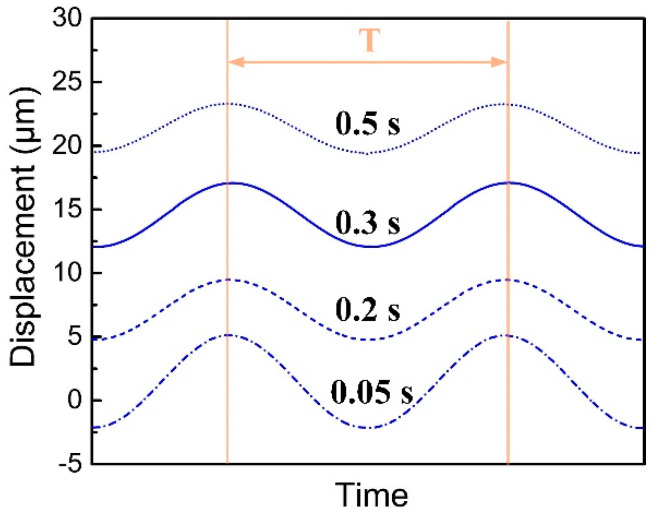
Displacement of Node 582 on the welding interface (No.1 scheme: amplitude, 35 µm, trigger force 200 N, plunging speed 0.3 mm/s, welding time 0.5 s).

Figure 7 presents the time–displacement curves (two vibration cycles) of the normal displacement (perpendicular to the welding surface) at the 582 node of No. 1 scheme. It can be seen that the average value of displacement response increases with the progress of welding, which is caused by the continuous pressing of the horn during the welding process, and the time–displacement curves present a harmonic state with the same frequency as the external excitation. In addition, the amplitude of displacement response decreases gradually.

**Figure 8:**
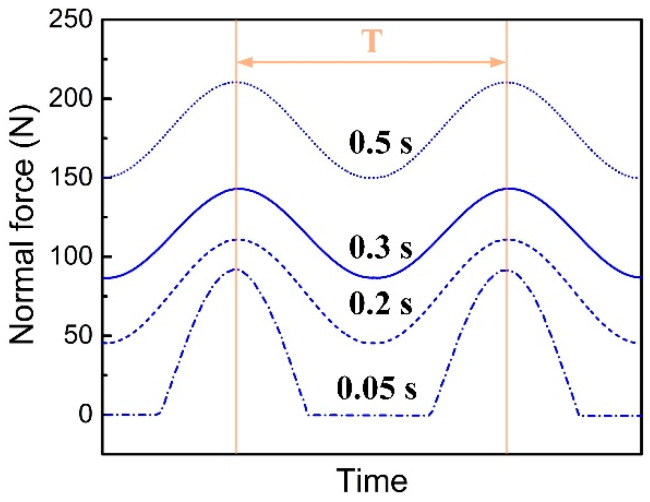
The Normal force of Node 582 (No.1 scheme: amplitude, 35 µm, trigger force 200 N, plunging speed 0.3 mm/s, welding time 0.5 s).

The normal force at the welding interface node 582 is also calculated by numerical simulation. Figure 8 shows normal force at different times (each time interval is assumed to be a steady-state response) of the No. 1 scheme. This shows that at the initial vibration stage, the welding interface is separated for a certain time, and the overall fluctuation of the curve is large. With the progress of the welding process, there is no separation state after 0.2 s, and the normal force increases gradually. That is to say that due to the small plunging distance of the horn in the early stage, there may be separation at the welding interface, which makes the normal force show nonlinear characteristics. However, with the increase of the plunging movement of the horn, the interface separation state no longer appears.

### 4.2. Temperature Characterization at the Welding Interface

**Figure 9:**
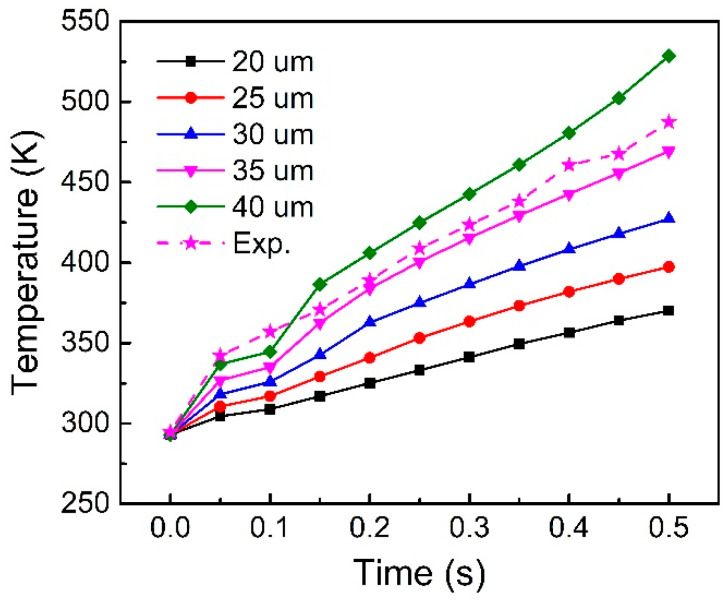
Node temperature on the welding interface under different vibration amplitudes.

For UPW, amplitude is one of the most important parameters. The amplitude will directly affect the vibration of the whole workpiece, which plays a decisive role in the heating process at the welding surface. Figure 9 shows the effect of amplitude on the temperature rise curve of the 582 node. The trend of the five curves shows that the heating rate increases significantly with the increase of amplitude. With the same welding time, a larger amplitude will result in a higher temperature. The experimentally measured result is also presented in Figure 9. It can be seen that the measured temperature is a little higher than the calculated one. The error of material parameters and the micro-sliding of the thermocouple during the welding contribute to the deviation between the calculated and measured results. However, the overall trend of experimental and calculated results are in good agreement.

**Figure 10:**
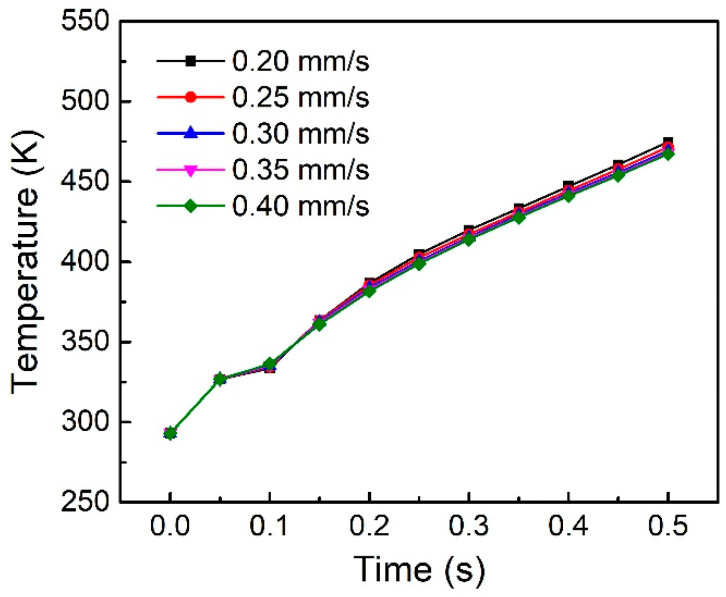
Node temperature on the welding interface under different plunging speeds.

Figure 10 shows the effect of plunging speed on the node temperature of the welding surface. The results show that reducing plunging speed can improve the temperature rise rate in the welding surface to a certain extent. However, its effect is not as great as that of amplitude. The reason for this phenomenon is that the faster plunging speed makes the welding force too large (far greater than the trigger force) in the whole welding process, which can be equivalent to increasing the stiffness of the structure, and reducing the dynamic strain of the structure, resulting in the reduction of heat generation to a certain extent.

**Figure 11:**
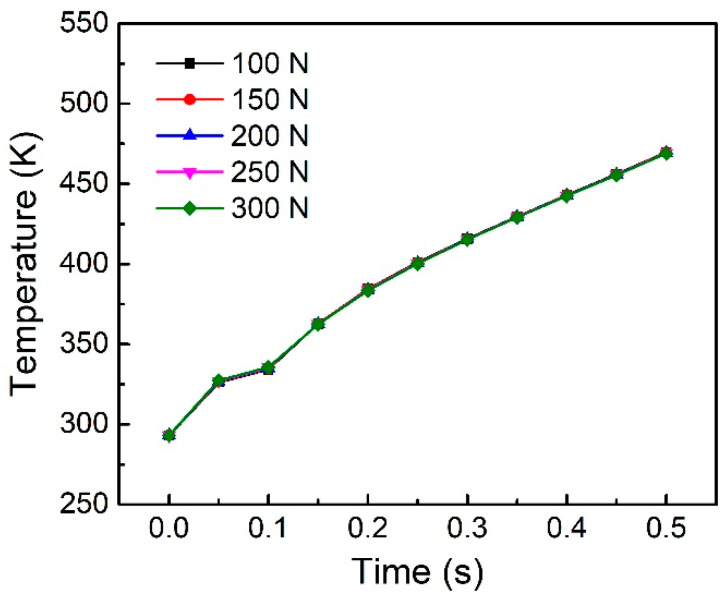
Node temperature on the welding interface under different trigger force.

At present, there is little analysis on the influence of trigger force on the welding temperature. Figure 11 shows the effect of trigger force on the welding temperature. It can be seen that the trigger force has no effect on the node temperature on the welding surface.

**Figure 12:**
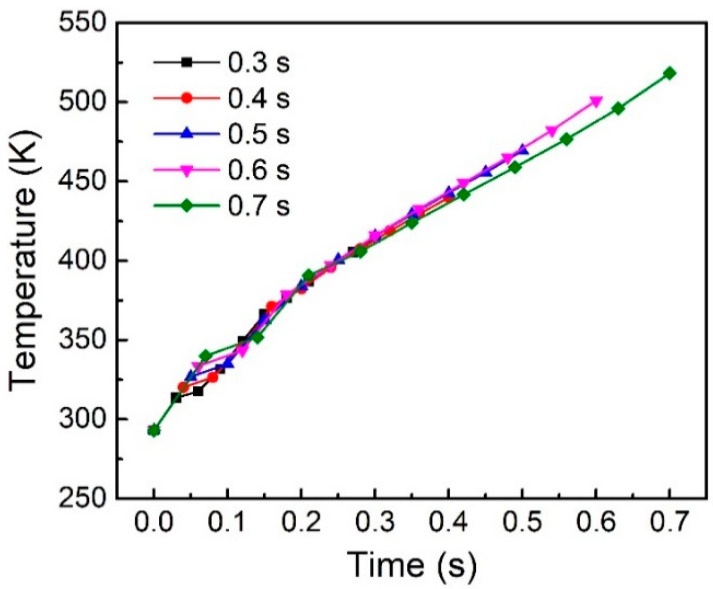
Node Temperature on the welding interface under different welding time.

When the amplitude, plunging speed and trigger force are fixed, the welding time will determine the amount of input welding energy. Figure 12 shows the effect of welding time on the node temperature on the welding surface. It can be seen that the temperature curves are roughly linearly related to the welding time. Longer welding time can make the welding area reach a higher temperature.

### 4.3. Heating Generation Evolution

**Figure 13:**
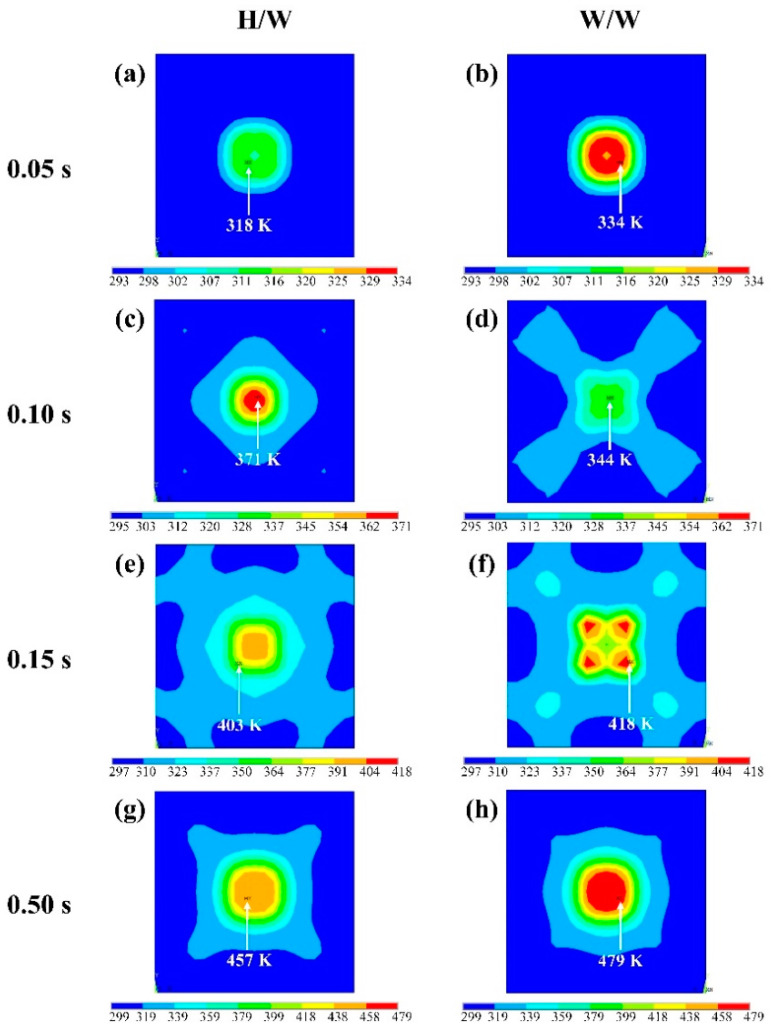
The temperature distribution on the horn/workpiece (H/W) interface and the workpiece/workpiece (W/W) interface at welding time of: (**a**,**b**) 0.05 s; (**c**,**d**) 0.10 s; (**e**,**f**) 0.15 s; (**g**,**h**) 0.50 s. (No.1 scheme: amplitude, 35 µm, trigger force 200 N, plunging speed 0.3 mm/s, welding time 0.5 s).

Figure 13 shows the temperature distribution on the horn/workpiece (H/W) interface and the workpieces interface (W/W), these show that the interface temperature distribution undergoes complex changes during the welding process when selecting scheme No. 1 for numerical analysis. Figure 13a,c,e,g show the temperature distribution of the H/W interface, and Figure 13b,d,f,h show the temperature distribution of the W/W interface. It can be seen from Figure 13 that at the initial time of 0.05 s, the highest temperature appears at the non-center of the welding area. This is because there is more sliding friction at the edge of the welding area at the initial stage, so this position becomes the location of the highest temperature. At 0.1 s, the highest temperature appears at the center of the H/W interface rather than the W/W interface. With the progress of welding, when the time is 0.15 s, the highest temperature returns to the W/W interface. Combined with the heat generation value, the friction heat generation has been reduced. When the time reaches 0.5 s, the highest temperature appears in the W/W interface, and the temperature at the H/W interface is also high, which is also the reason why the upper surface of the workpiece will melt after welding, especially when the welding time is long.

**Figure 14:**
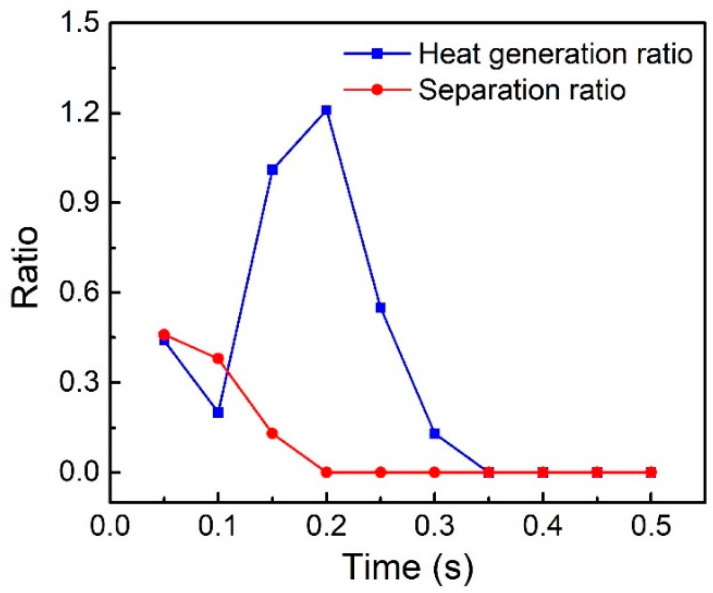
Heat generation ratio and separation ratio.

Figure 14 shows changes in the heat generation ratio (the ratio of friction heat generation to viscoelastic heat generation), and separation ratio (the time proportion of nodes to separation in a vibration cycle) with welding time. When the welding time is less than 0.2 s, friction heat is dominant and the proportion of friction heat gradually increases. This is because when the welding time is less than 0.2 s, the separation ratio of the two workpieces decreases, which enhances frictional heat generation. When the welding time exceeds 0.2 s, the two workpieces no longer separate, and there is no friction heat generation.

## 5. Conclusions

In this paper, the thermal-mechanical coupling analysis of UPW is carried out based on the HBM. The contact behavior and temperature characterization on the welding surface are analyzed. The numerical results indicate that there are two states of contact and separation at the welding interface in the early stage of welding, and the separation state gradually disappears with the progress of welding. The contact force gradually increases with the plunging process of the horn. The amplitude and welding time have important influence on the temperature of the welding surface, while the influence of the plunging speed is relatively limited, and the trigger force has almost no influence. Increasing amplitude and welding time can significantly increase the welding surface temperature. In the early stage of welding, as the separation ratio of the workpieces decreases, the frictional heat generation gradually increases and is greater than the viscoelastic heat generation; while in the middle and late stages of welding, the workpiece adheres and the frictional heat generation disappears.

## Figures and Tables

**Table 1:** The numerical scheme of ultrasonic welding of CF/PA6.

Number	Amplitude/µm	Trigger Force/N	Plunging Speed/mm·s^−1^	Welding Time/s
1	35	200	0.30	0.5
2	20	200	0.30	0.5
3	25	200	0.30	0.5
4	30	200	0.30	0.5
5	40	200	0.30	0.5
6	35	100	0.30	0.5
7	35	150	0.30	0.5
8	35	250	0.30	0.5
9	35	300	0.30	0.5
10	35	200	0.20	0.5
11	35	200	0.25	0.5
12	35	200	0.35	0.5
13	35	200	0.40	0.5
14	35	200	0.30	0.3
15	35	200	0.30	0.4
16	35	200	0.30	0.6
17	35	200	0.30	0.7

**Table 2:** Materials parameters of CF/PA6 for the simulation.

Properties	Value
Density (kg·m^−3^)	1290
Specific heat (J·kg^−1^·K^−1^)	1950
Thermal conductivity (W·m^−1^·K^−1^)	13.4
Poisson ratio	0.44
Friction coefficient	0.1

## Data Availability

Data is contained within the article.
